# Loneliness and meaning in life are reflected in the intrinsic network architecture of the brain

**DOI:** 10.1093/scan/nsz021

**Published:** 2019-03-29

**Authors:** Laetitia Mwilambwe-Tshilobo, Tian Ge, Minqi Chong, Michael A Ferguson, Bratislav Misic, Anthony L Burrow, Richard M Leahy, R Nathan Spreng

**Affiliations:** 1 Montreal Neurological Institute, Department of Neurology and Neurosurgery, McGill University, Montreal, QC, Canada; 2 Psychiatric and Neurodevelopmental Genetics Unit, Center for Genomic Medicine, Massachusetts General Hospital, Boston, MA, USA; 3 Department of Electrical Engineering-Systems, University of Southern California, Los Angeles, CA, USA; 4 Beth Israel Deaconess Medical Center, Harvard Medical School, Boston, MA, USA; 5 Department of Human Development, Cornell University, Ithaca, NY, USA; 6 Departments of Psychiatry and Psychology McGill University, Montreal, QC, Canada

**Keywords:** resting-state functional connectivity, partial least squares, individual differences, personality

## Abstract

Social relationships imbue life with meaning, whereas loneliness diminishes one's sense of meaning in life. Yet the extent of interdependence between these psychological constructs remains poorly understood. We took a multivariate network approach to examine resting-state fMRI functional connectivity’s association with loneliness and meaning in a large cohort of adults (N = 942). Loneliness and meaning in life were negatively correlated with one another. In their relationship with individually parcelled whole-brain measures of functional connectivity, a significant and reliable pattern was observed. Greater loneliness was associated with dense, and less modular, connections between default, frontoparietal, attention and perceptual networks. A greater sense of life meaning was associated with increased, and more modular, connectivity between default and limbic networks. Low loneliness was associated with more modular brain connectivity, and lower life meaning was associated with higher between-network connectivity. These findings advance our understanding of loneliness and life meaning as distinct, yet interdependent, features of sociality. The results highlight a potential role of the default network as a central hub, providing a putative neural mechanism for shifting between feelings of isolation and purpose.

## Introduction

Loneliness and life meaning are psychologically-bound constructs closely tied to sociality (Twenge *et al.,* 2003; Stillman *et al.,* 2009; Lambert *et al.,* 2013). As a social species, humans typically seek out social bonds and search for meaning and purpose throughout the life-course. Indeed, both loneliness and a reduced sense of meaning are closely associated with declines in functional capacity (Perissinotto *et al.,* 2012), dementia onset (Boyle *et al.,* 2012; Holwerda *et al.,* 2014), and mortality in later life (Boyle *et al.,* 2009; Hill and Turiano, 2014; Holt-Lunstad *et al.,* 2015). Despite these psychological and functional relationships, loneliness and meaning in life (MIL) are considered to be distinct constructs and their degree of interdependence remains poorly understood. Loneliness reduces the perception of a meaningful existence (Stillman *et al.,* 2009)—the sense that life has purpose, significance, and coherence (Martela and Steger, 2016). This association appears to be reciprocal as MIL is strongly associated with the presence of close relationships (Klinger, 1977; Ebersole, 1998), and previous reports show that the subjective perception of a meaningful life promotes social engagement and helps sustain close social bonds (Stillman and Lambert, 2013; Steptoe and Fancourt, 2019). Loneliness arises due to deficiencies in the quality or quantity of social ties and the absence of social connectedness, in turn, diminishes MIL, suggesting that this relationship may also be reinforcing (Baumeister and Leary, 1995). But are these constructs opposite sides of the same coin, or are they emergent from distinct mechanisms?

Loneliness is characterized by implicit hyper-vigilance for social threats (Cacioppo *et al.,* 2016). While this can facilitate the identification of viable social partners and prevent rejection, prolonged loneliness shifts exogenous attentional processes towards perceived social threats (Bangee *et al.,* 2014; Cacioppo *et al.,* 2015). Altered attention to external stimuli may affect how individuals internalize perceived information and make endogenous judgments about MIL (Hicks *et al.,* 2010). Externally-and internally-guided cognitive processes are mediated by different neural networks and their interactions (Corbetta and Shulman, 2002; Spreng *et al.,* 2010). This raises the possibility that loneliness and MIL are dissociable at the level of the brain, and subserved by distinct brain networks. Investigating how individual differences in loneliness and MIL are reflected within these neurocognitive systems may advance our understanding of their interdependence, and how they interact to guide adaptive and maladaptive behaviors.

A growing body of neuroimaging studies have provided important insights into the neural correlates of loneliness, reflecting changes in brain regions associated with processing of social information. In a task-based functional magnetic resonance imaging (fMRI) study, lonely individuals showed increased bilateral activation in the visual cortex in response to unpleasant social images compared to unpleasant non-social images. Regions implicated in reward processing (e.g. ventral striatum, amygdala) and perspective-taking (e.g. temporoparietal junction) showed lower activation when positive social images were presented, suggesting that lonely individuals may derive less pleasure from rewarding social stimuli (Cacioppo and Hawkley, 2009). Furthermore, other studies have linked loneliness to changes in brain morphology within the default network (DN), a neural system involved in social and self-related processes (Andrews-Hanna *et al.,* 2014). Loneliness is negatively correlated with grey matter volume (Kanai *et al.,* 2012) and white matter density (Nakagawa *et al.,* 2015) in the left posterior superior temporal sulcus (pSTS). These findings indicate that loneliness may compromise the structural and functional integrity of multiple brain regions.

Resting-state functional connectivity (RSFC) has been an invaluable analytic approach for investigating the functional interactions between anatomically separate brain regions and their relationship with behavior (Stevens and Spreng, 2014). Unlike task-based fMRI paradigms, resting-state functional magnetic resonance imaging (rs–fMRI) is task-free and can be used to simultaneously identify multiple functional networks correlated with behavior. Furthermore, previous analyses of rs-fMRI data from healthy adult populations have consistently shown strong congruence between brain networks derived from resting-state and those from task-based studies (Cole *et al.,* 2014; Stevens and Spreng, 2014; Tavor *et al.,* 2016).

Prior studies have used rs-fMRI to characterize intrinsic functional brain networks related to loneliness and MIL. Greater feelings of loneliness have been associated with less integrated connectivity between attention networks (Tian *et al.,* 2017), as well as increased RSFC within the cingulo-opercular network, which is implicated in cognitive control (Layden *et al.,* 2017). These intrinsic changes are consistent with behavioral reports of associations between hyper-vigilance and loneliness (Cacioppo *et al.,* 2016). An investigation of the neural basis of meaning (Waytz *et al.,* 2015) reported increased connectivity among regions of the medial temporal lobe subsystem of the DN, implicated in autobiographical remembering and mental simulation (Andrews-Hanna *et al.,* 2014). While loneliness and MIL are correlated at the level of behavior, the analytical approaches used to characterize the neural representation of each construct have focused on functional connectivity of select brain regions or networks of interest, thus precluding inferences on a whole-brain level of integrated networks that can provide insight regarding the relationship between loneliness and MIL. Here, we investigate individual differences in the neural representation of loneliness and MIL within a single analytical framework.

The goal of the present study was to assess how whole-brain RSFC is associated with individual differences in loneliness and MIL. We characterized the intrinsic architecture of brain connectivity within a large population of healthy young adults using RSFC and individually parcellated brain regions (Chong *et al.,* 2017), respecting that the localized topology varies across individuals in the cortex (e.g. Stevens *et al.,* 2015) in order to identify the pattern of functional connectivity within and between large-scale networks. Using multivariate partial least squares (PLS), we characterized how patterns of RSFC relate to individual differences in perceived loneliness and MIL. This approach permits both replication of previous RSFC patterns, and exploratory examination of behavioral associations outside previously examined networks.

By examining the intrinsic functional connectivity underlying individual differences in loneliness and MIL, we test two hypotheses: First, loneliness would be associated with greater connectivity between regions that support attention, including the FPN, dorsal attention (DAN), and the ventral attention networks (VAN; Corbetta and Shulman, 2002). In contrast, MIL would be associated with greater connectivity within the DN. Our second hypothesis was that these patterns of RSFC would be inversely related (i.e. individuals with high levels of loneliness will share the same pattern of brain connectivity as those with a low sense of MIL and vice-versa). If confirmed, this would provide support for theoretical models of sociality suggesting that loneliness and MIL are distinct yet interdependent constructs (Lambert *et al.,* 2013).

## Methods

### Participants

Participant data were collected as part of the Human Connectome Project (HCP) 1200 subject release dataset (http://www.humanconnectome.org). Participants were excluded if they did not meet the following criteria: (i) completed all rs-fMRI scans (REST1 and REST2); (ii) completed all relevant neuropsychological testing for emotional well-being; (iii) participants with a score of 26 or below on the Mini Mental Status Examination (MMSE)—which could indicate marked cognitive impairments. Investigations of individual differences require large samples for adequately powered analyses. Assuming a typical correlation of approximately .25 between brain and behavior (e.g. Hemphill, 2003), a sample of more than 120 is recommended in order to have 95% confidence that a correlation is greater than zero. A total of 942 healthy adults were included in the current study (53% female; mean age: 28.04; age range: 22–37). Table 1 shows the sample demographics.

**Table 1:** Sample Demographics

Gender			
	*n*	*%*	
*Female*	*506*	*53.7*	
*Male*	*436*	*46.3*	
Variable	*Mean*	*s.d.*	*Range*
Age	28.04	3.45	23–37
Loneliness	50.97	8.51	37.6–82.9
Meaning & Purpose	51.91	8.73	29.4–71.6
MMSE	29.05	0.99	23–30
Neuroticism	16.42	7.34	0–43
Extroversion	30.73	6.04	10–47
Agreeableness	32.12	4.95	13–45
Conscientiousness	34.56	5.91	11–48
Openness	28.33	6.26	10–47
Positive Affect	50.22	7.83	21.9–71.6

### Behavioral Measures

Behavioral assessments of social relationships and psychological well-being in the HCP sample included were obtained using the unadjusted scaled scores (t-scores) from the NIH Toolbox Emotion measures (http://www.nihtoolbox.org). All behavioral measures were treated as a continuous variable, and any references to high or low scores made are based on our specific sampling distribution.

#### Assessment of loneliness

Loneliness was assessed using the Loneliness survey from the NIH Toolbox on Emotion. This 5-item questionnaire is composed of items taken from a psychometrically validated assessment of loneliness (Salsman *et al.,* 2013). Participants were presented with statements such as ‘I feel alone and apart from others’ (1 = Never; 2 = Rarely; 3 = Sometimes; 4 = Usually; 5 = Always).

#### Assessment of meaning in life

MIL was assess using the Meaning and Purpose survey from the NIH Toolbox on Emotion. This 18-item questionnaire is composed of items taken from psychometrically validated assessments of meaning and purpose (Salsman *et al.,* 2013), and examines the extent to which people feel like their lives matter and make sense. An example item is, ‘I have a good sense of what makes my life meaningful’ (1 = Strongly disagree; 2 = Disagree; 3 = Neither agree nor disagree; 4 = Agree; 5 = Strongly agree).

#### Assessment of personality and positive affect

Neuroticism and extroversion have been previously shown to mediate the relationship between loneliness and dorsolateral prefrontal cortex (Kong *et al.,* 2015), whereas personality and positive affect influence people’s perception of MIL (King *et al.,* 2006). Therefore, to assure the specificity of our findings, we controlled for these select covariates during our analysis. Personality measures of neuroticism, extroversion, openness, agreeableness, conscientiousness, were assessed using the 60-item version of the NEO-Five Factor Inventory. The NIH Toolbox Positive Affect Survey was used to assess participants’ levels of positive affect during the past seven days. Participants were presented with statements such as ‘I feel cheerful’ (1 = Not at all; 2 = A little bit; 3 = Somewhat; 4 = Quite a bit; 5 = Very much).

### Resting-state Functional Connectivity

The rs-fMRI data from the HCP was used for this study. The rs-fMRI runs were acquired for a total of 1 hour over the course of two sessions. For more details of the scan parameters, see Smith *et al.,* (2013). Scans were processed using the HCP minimal preprocessing pipeline, which includes normalization to the MNI-152 template (Glasser *et al.,* 2016). FIX ICA cleaned data was used for analysis (Glasser *et al.,* 2016).

To identify functional networks, we parcellated the cortex into 400 functionally-defined regions for each individual separately. We refined the initial group parcellation developed by Schaefer *et al.,* (2018) so that for each subject the parcel boundaries are optimized with respect to that subject’s rs-fMRI (Chong *et al.,* 2017). Initialization with a common parcellation results in automatic correspondence between parcels across subjects. By using a group sparsity constraint to model connectivity, we leveraged group similarities in connectivity between parcels while optimizing their boundaries for each individual. We applied this approach with initialization across the entire cohort in groups of 20 unrelated participants. Prior work on validating this approach showed improved homogeneity of resting activity within the refined parcels (Chong *et al.,* 2017). Additionally, comparisons with task-based localizers showed a consistent reduction of variance of statistical parametric maps within the refined parcels relative to the group-based initialization indicating improved delineation of regions of functional specialization. This method enables a more accurate estimation of individual functional areas while maintaining consistency across individuals with a standardized topological atlas (Chong *et al.,* 2017). Each parcel was matched to a corresponding network in the 7 network parcellation by Yeo *et al.,* (2011), which consisted of the visual, somatomotor, dorsal attention, ventral attention, limbic, frontoparietal, and default networks. For each participant, BOLD time-series for the two 15-min rs-fMRI scans within each session were temporally standardized (subtracted the mean and divided by standard deviation) and concatenated. The Pearson correlation coefficient between each pair of vertices was computed. The correlation coefficient matrix was then spatially standardized and averaged within and across parcels, resulting in a 400 x 400 functional connectivity matrix (Ge *et al.,* 2017). The two connectivity matrices computed from the two sessions for each participant were averaged.

### Behavioral Data Analysis

Three sets of analysis were performed to examine the behavioral relationship between loneliness and MIL in Python (https://www.python.org/). In our first analysis, we used the t-scores for the self-report behavioral measures and calculated the Pearson correlation coefficient between each measures. This also allowed us to determine whether loneliness and MIL were inversely related to one another using the NIH-emotion scales. We also examined this association controlling for covariates (age, gender, MMSE, positive affect, and personality measures) using partial correlation. Finally, t-tests were conducted to identify possible gender differences in the distribution of scores between loneliness and MIL, as well as in covariates of interest. Statistical significance was set at *P* < 0.05.

### Partial Least Squares Analysis

PLS was performed to quantify RSFC related to individual differences in loneliness and MIL. PLS is a multivariate statistical technique which uses a data-driven approach to directly measure brain-behavior relationships (McIntosh and Lobaugh, 2004; McIntosh and Mišić, 2013). We chose this method of analysis because it allowed for inferences about individual differences in the intrinsic connectivity of large-scale neurocognitive networks. PLS identifies linear combinations of the original variables (functional connections and behavioral measures) that maximally covary with each other across participants. The resulting patterns (termed latent variables or LVs) can be interpreted as optimally-paired functional networks and behavioral phenotypes, respectively.

In the present study, we used PLS to examine the relationship between RSFC, loneliness, and MIL. Two matrices were computed for this analysis. The **X** matrix was organized such that the parcellated functional connectivity matrix for each participant was concatenated, resulting in a 942 x 400 x 400 matrix. The **Y** matrix consisted of the individual scores for loneliness and MIL for all participants, creating a 942 x 2 matrix. The **X** and **Y** matrix were centered and normalized across participants. Singular value decomposition of the cross-correlation matrix **X’Y** yields several mutually-orthogonal LVs, each composed of three elements: (i) a left singular vector, containing weights for each of the behavioral measures; (ii) a right singular vector, containing weights for each of the functional connections; and (iii) a scalar singular value. Squared singular values reflect effect size: they are proportional to the covariance between connectivity and behavior that is accounted for by each latent variable. The number of latent variables is equal to the rank of **X’Y**; in the present case, this is the number of behavioral measures (ii).

The significance and reliability of each LV were evaluated in permutation testing and bootstrap resampling, respectively. We first assessed the significance of the pattern of functional connectivity captured by a given LV using permutation tests to determine how different the results are from chance. To do this, 500 permutation tests were computed in which the order of the rows of one of the data matrices (**X**) was randomly rearranged. Columns of the permuted matrix are then correlated with the behavioral matrix **Y** and the correlation matrix is subjected to singular value decomposition as described above. This process generates a distribution of singular values under the null hypothesis that there is no relationship between functional connectivity and behavior. The significance of the LV is estimated by computing the proportion of times the permuted singular values (covariance explained) is higher than the observed singular values (significance thresholded at *P* < .05).

To assess the reliability of weights for individual connections and behavioral measures, we used bootstrap resampling. The rows of both data matrices (**X** and **Y**) were sampled with replacement and a resampled correlation matrix (**X’Y**) was re-computed. The matrix was subjected to singular value decomposition and the process was repeated 500 times to estimate a sampling distribution for each singular vector (i.e. connection and behavior) weight. To identify connections and behaviors that (a) make a large contribution to the overall multivariate pattern and (b) are relatively insensitive as to who is in the sample, we calculated the ratio between each weight and its bootstrap-estimated standard error. The resulting ‘bootstrap ratios’ (BSRs) are large for connections/behaviors that have large weights and narrow confidence intervals. If the sampling distribution is approximately unit normal, BSRs are equivalent to z-scores. Brain network connections were considered reliable if the absolute value of the BSR > 2 (approximately P < .05) and were visualized using BrainNet Viewer (Xia *et al.,* 2013). To account for potential confounds, multiple regression analysis was performed on the brain connectivity scores with behavioral scores controlling for age, gender, personality measures, and positive affect.

We also examined the extent to which network-level functional connectivity contributes to individual differences in behavior. To quantify the network-level contributions to the connectivity pattern identified by the PLS analysis, two separate weighted adjacency matrices were constructed reflecting the positive and negative PLS weights, respectively. The nodes of the graph represent the 400 brain regions defined by the individual parcellation scheme, and the edges represent the BSR weight for each pairwise connection. The matrices were thresholded such that BSRs with an absolute value less than 2 were set to 0. Positive BSRs greater than 2 were set to 1, and negative BSRs less than −2 were set to −1. The network-level functional connectivity contributions were quantified by averaging the weights of all connections in a given network, thus generating a 7 x 7 matrix. Next, permutation testing was applied on the full thresholded matrix by randomly re-ordering the network labels (preserving the number of nodes originally assigned to each network) and re-calculating the network means 1000 times to build a sampling distribution under the null that network assignment does not contribute to the connectivity pattern. The significance of the pairwise connections of the original 7 x 7 matrix was determined by estimating the proportion of times the values of the sampling distribution were greater than or equal to the original value (Shafiei *et al.,* 2018).

### Modularity Analysis

To further characterize the pattern of connectivity identified by the PLS analysis, we quantified modularity, a global network measure that estimates how well a network can be divided into modules (or communities) with stronger within-module than between-module connections (Girvan and Newman, 2002). Modular organization within a network is as a metric of efficient information processing and relates to functional specialization (Bullmore and Sporns, 2009). The modularity measure *Q(p)* for a given partition *p* of a graph *G* can be defined as the proportion of edges in *G*, that fall within the same module, subtracted from the proportion of edges that would be expected by chance. The objective of this modular algorithm is to identify the partition *p* that maximizes *Q.* A modularity value of *Q =* 0 is expected if the edges of a graph were formed randomly, while a graph with a *Q >* 0.3 is generally an indicator of significant modular structure (Newman and Girvan, 2004). There are multiple methods for identifying modules, however, and here we used an *a priori* mapping of nodes to the network modules defined by Yeo *et al.,* (2011). This allowed us to quantify the strength of segregation of functional networks. We sub-divided the thresholded PLS connectivity matrix into two separate graphs: one containing just positive PLS weights and the other the negative PLS weights. Graph theoretical analyses were performed using functions implemented using the Brain Connectivity Toolbox (Rubinov and Sporns, 2010). Network modularity estimates were computed with a Louvain-like fast-unfolding algorithm (Blondel et al. 2008), using the average modularity across 1000 runs of the algorithm.

## Results

### Descriptive data analysis

Sample characteristics for age, gender, loneliness scores, meaning in life scores, MMSE scores, personality scores, and positive affect are displayed in Table 1. Pearson correlation between these behavioral measures revealed a negative correlation between loneliness and meaning in life (*r*(940) = −.45, *P* < .001, 95% CI = [0.53, 0.36]), which supports previous findings (Stillman *et al.,* 2009). Loneliness was also negatively correlated with extroversion (*r(*940*)* = −.42, *P* < .001, 95% CI = [−0.50, −0.32]), agreeableness (*r(*940*)* = −.27, *P* < .001, 95% CI = [−0.36, −0.17]), and conscientiousness (*r(*940*)* = −.32, *P* < .001, 95% CI = [−0.41, −0.22]), and positive affect (r(940) = −.47, *P* < .001, 95% CI = [−0.55, −0.39]); and positively correlated with neuroticism (*r(*940*)* = .57, *P* < .001, 95% CI = [0.49, 0.64]) and openness (*r(*940*)* = .08, *P* = .01, 95% CI = [−.02, 0.18]). MIL was negatively correlated with neuroticism (*r(*940*)* = −.43, *P* < .001,95% CI = [−0.51, −0.34]) and gender (*r(*940*)* = −.09, *P* = .01, 95% CI = [−0.19, 0.01]), and positively correlated with extroversion (*r(*940*)* = .40, *P* < .001, 95% CI = [0.31, 0.49]), agreeableness (*r(*940*)* = .25, *P* < .001, 95% CI = [0.15, 0.34]), conscientiousness (*r(*940*)* = .35, *P* < .001, 95% CI = [0.26, 0.44]), and positive affect (*r(*940*)* = .52, *P* < .001, 95% CI = [0.43, 0.59]). No other significant correlations were noted between covariates.

Analyses were also conducted to determine any gender differences in behavioral measures. The means and standard deviations for loneliness, MIL, personality traits, and positive affect by gender are displayed in Supplementary Table 1. While there was no significant gender differences for loneliness in our sample, *t*(940) = 0.34, *P* = .73, *d* = 0.02, female participants reported higher meaning in life scores (M = 52.62, s.d. = 8.69) than male participants (M = 51.09, s.d. = 8.71), *t*(940) = 2.70, *P* < .01, *d* = 0.18).

### Intrinsic functional connectivity results

**Fig. 1:**
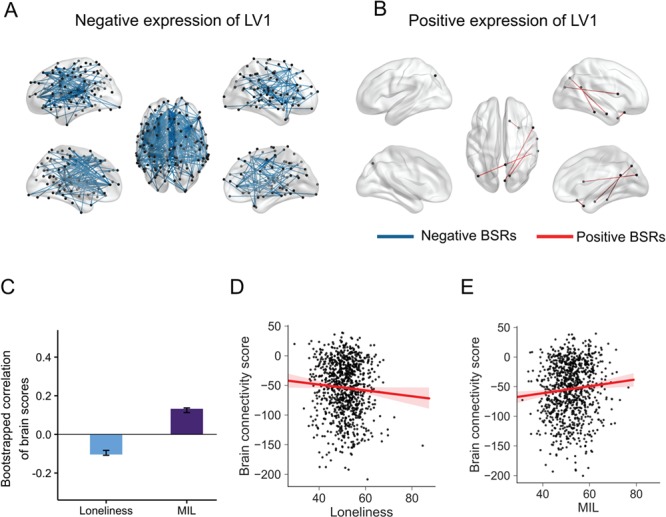
Behavioral PLS results. Analysis revealed one significant latent variable (LV). The functional connections that most reliably express the brain/behavior correlations thresholded at 95% bootstrap ratio. The pattern of connectivity for LV1 depicted in (A) blue represent the connectivity weights for LV1 that covary negatively with loneliness, while those in (B) red covary positively with meaning in life (MIL). The top 2% connections are shown for each. (C) Correlations between participants’ brain connectivity scores and behavioral measures for LV1. Error bars indicate the 95% confidence intervals derived from the bootstrap estimate. Scatter plots show the relationship captured by the PLS analysis for individual brain connectivity scores corrected for age, gender, positive affect, and personality measures as a function of loneliness (D) and MIL (E).

We first examined the multivariate relationship between RSFC, loneliness, and MIL using behavioral PLS. The analysis identified one significant pattern of connectivity that reliably expressed individual differences in loneliness and MIL (loneliness *r* = −.10; MIL *r* = .13; permuted *P* = .01; 17.6% covariance explained). Loneliness was found to negatively correlate with the pattern of brain connectivity of LV1, whereas MIL correlated positively with this pattern of brain connectivity (Figure 1C). To assure the specificity of these results, a partial correlation analysis was used to test whether the relationship between behavioral measures and brain connectivity scores remained significant after controlling for age, gender, personality, and positive affect. The results remained significant for loneliness (*pr*(932) = −.08; *P* = .01, 95% CI [−0.14, −0.01]) and MIL (*pr*(932) = .09, *P* = .003, 95% CI [0.03, 0.16]).

Figure 1A-B shows reliable ROIs that covary with each other. The edges connecting the nodes for the negative and positive dimension of LV 1 represent the top 2% BSR weights. Overall, the connectivity pattern for the negative expression of LV 1 showed densely interconnected nodes when compared to that of the positive expression. Participants with high levels of loneliness showed extensive between-network connectivity across the brain. Specifically, nodes located within DN, SOM, and FPN were highly interconnected. In addition, functional connectivity was observed between bilateral regions in the visual network with the frontal and parietal operculum. In contrast, high levels of MIL correlated with increased functional connectivity between regions involving the DN and limbic network. This included bilateral connectivity between posterior parietal regions, as well as with nodes located in the anterior regions of the FPN.

**Fig. 2:**
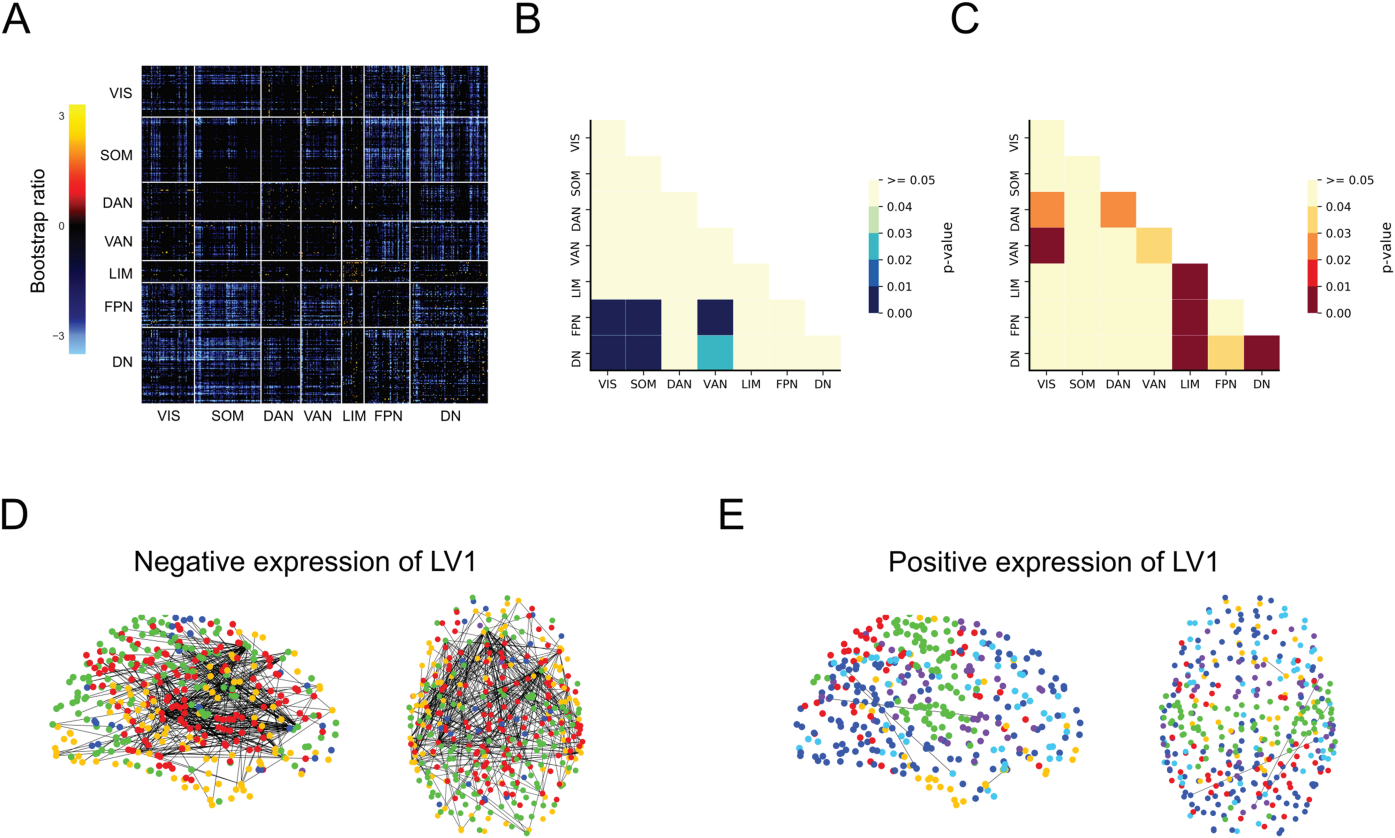
Functional network organization. (A) The correlation matrix of reliable pairwise connections associated with loneliness and meaning in life (MIL; thresholded bootstrap ratio ± 2.0 to 3.5). Significant contributions of resting-state network pairs to the connectivity pattern for the (B) negative expression of the first latent variable (LV1) and (C) positive expression of LV1. Sagittal and axial views of the resting-state functional connectivity pattern associated (D) loneliness and (E) MIL. The colors indicate the nodes that belong to the same module and node size is proportional to the number of edges connecting it to the network. VIS = visual; SOM = somatomotor; DAN = dorsal attention; VAN = ventral attention, LIM = limbic, FPN = frontoparietal network; DN = default network.

The PLS analysis identified reliable connectivity patterns that explain individual variability in loneliness and MIL. However, from the results of this analysis alone, it is difficult to gauge whether certain networks contribute more to the overall network connectivity pattern than others. To address this question, we used permutation testing on the functional covariance matrix representing the pairwise BSRs for each of the 400 brain regions (Figure 2A) to examine the relative within and between network contributions of the seven networks defined by the parcellation scheme. As shown in Figure 2B, the strongest contributions to the RSFC pattern associated with the negative expression of LV1 were from the DN and FPN. Specifically, between network connections of both the DN and FPN with VIS, SOM, and VAN were found to contribute significantly to the overall connectivity pattern (DN: VIS = *P* < .001; SOM = *P* < .001, and VAN = *P* < .05; FPN: VIS = *P* < .001; SOM = *P* < .001, and VAN = *P* < .001). For MIL, we found that both between and within network connectivity contributed to the RSFC pattern (Figure 2C). The pairwise connections that contributed the strongest were between the DN with the LIM (*P* = .001) and FPN (*P* < 0.05); the FPN and the VIS (*P* < .05) and LIM (*P* = .001); and between the VIS and the DAN (*P* < .05) and VAN (*P* < .01). As for the within network connectivity, the DAN (*P* < .05), VAN (*P* < .05), LIM (*P* < .001), and DN (*P* < .01) were found to contribute significantly to the RSFC pattern related to MIL.

### Gender control analysis

To account for possible effects of gender, an ANCOVA was conducted to examine the effects of gender on the PLS brain scores while controlling for age, personality, and positive affect. We found that there was a significant effect of gender, *F*(1,933) = 30.48, *P* < .001, partial eta squared = .032. We then reanalyzed the data to assess the relationship between RSFC, loneliness, MIL, and gender. In the group analysis using PLS, the brain-behavior correlation for both groups co-varied together. Critically, no gender interaction was observed (see Supplementary Figure 1). This suggests that the magnitude of the association is weaker in women. However, the associations with functional connectivity are still significant when controlling for gender, in addition to neuroticism, extroversion, agreeableness, conscientiousness, openness, positive affect and age; for loneliness [pr(932) = −.08, *P* < .05] and MIL [pr(932) = .10, *P* < .005].

### Modularity

**Fig. 3:**
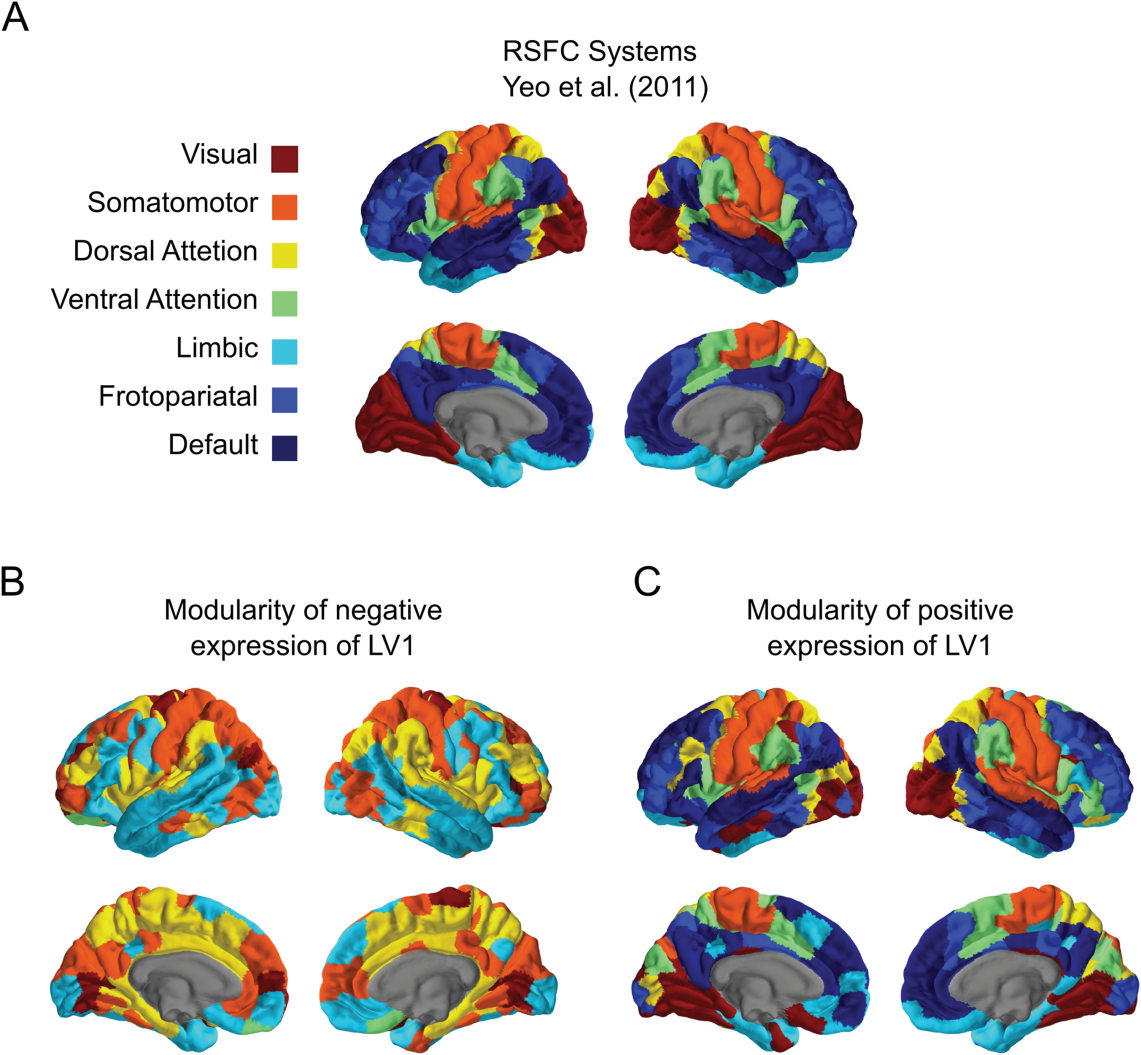
Cortical surface maps of the resting-state functional connectivity (RSFC) modules. (a) the modular organization for RSFC defined by 7 network parcellation of Yeo *et al.,* (2011). Modular organization of the connectivity pattern associated with (b) high-loneliness/low-meaning in life and (c) high-meaning in life/low-loneliness. Color-coding brain regions according the module assignment in (a).

Having established that cohesion within and between select networks appears to play an important role in the connectivity profiles underlying differences in behavior, we sought to investigate the global network organization of LV1 by assessing the modular structure of the connectivity pattern. The modularity for each pattern provides a metric for quantifying the segregation of functional networks, with higher *Q* indicating a stronger segregation of functional networks. Using a pre-defined partition based on the modules previously reported in Yeo *et al.,* (2011), we calculated the modularity quality index *Q* of the thresholded weighted graphs representing the negative and positive BSR weights. Graphical representations of the modular structure associated with each behavior are shown in Figure 2D-E (see Figure 3B-C for projections on the cortical surface). The features of community structure for loneliness and MIL differed in the number of communities detected and in the distinctiveness of these communities. While the algorithm used to examine the community structure revealed 7 modules for MIL that largely corresponded with the pre-defined partition, we identified only 5 modules for the connectivity pattern for loneliness. Specifically, nodes previously assigned to the FPN and DN appear to be integrated with parts within the SOM and VIS networks (Figure 3B). Next, we measured the mean *Q* to quantify the segregation of functional networks and found that loneliness was less segregated (mean *Q* = 0.15) relative to MIL (mean *Q* = 0.58). Taken together, these findings reflect that loneliness and MIL are characterized by differences in modular organization of brain networks.

## Discussion

Loneliness and meaning in life are important for guiding everyday behavior and sustaining mental health and well-being over the life course and into advanced age. Yet their neural signatures remain poorly understood. Here we used a multivariate analytical model to examine patterns of intrinsic functional connectivity associated with individual variability in loneliness and MIL in a large sample of healthy adults. There were three primary findings. First, we identified reliable patterns dissociating whole-brain RSFC related to individual differences in loneliness and MIL. Second, we observed a core role for default network connectivity in differentiating loneliness and meaning in life. While default and frontoparietal interactions, among others, were associated with higher levels of loneliness, this pattern differed for MIL where connectivity between default and limbic brain regions was associated with a greater sense of meaning. Finally, greater feelings of loneliness were associated with lower modularity, or increased integration, between the default and frontoparietal networks and more externally-oriented networks including somatosensory and visual brain regions. In contrast, a stronger sense of life meaning was associated with greater modularity among the limbic and default networks.

Current theoretical models of sociality suggest that loneliness and MIL are discrete yet interdependent, and potentially reinforcing (Twenge *et al.,* 2003; Stillman *et al.,* 2009; Lambert *et al.,* 2013). However, only a few studies have investigated the relationship between the loss of social functioning (i.e. loneliness) and MIL, and these have primarily employed behavioral methods (Lambert *et al.,* 2013; Stillman and Lambert, 2013). More recently, investigations into the intrinsic functional architecture of the brain at rest (i.e. in the absence of explicit task demands) have demonstrated that these durable features of brain organization can enhance our understanding of enduring features of mental function (Stevens and Spreng, 2014; Smith *et al.,* 2015). Here we leveraged this idea to explore patterns of functional connectivity associated with individual differences in loneliness and MIL.

We predicted that the DN, through its role in mediating internally directed cognition, would be associated with MIL. A greater sense of life meaning has previously been associated with increased connectivity within the medial temporal lobe subsystem of the DN (Waytz *et al.,* 2015). Our data complements this finding by showing increased connectivity within nodes of the DN associated with higher MIL. Additionally, we observed a robust, albeit unpredicted, pattern of connectivity within and between networks typically implicated in internally-directed cognitive processes associated with higher MIL, including the limbic and default networks, as well cognitive control regions of the FPN. The limbic network is involved in emotional processing, which involves monitoring, evaluating, and adjusting emotional reaction to align with current goals. Thus the ability to internally reflect upon one’s affective state, may be important for a sense of meaning, particularly when experiencing negative emotions (Kross and Ayduk, 2011). Consistent with this idea, individuals with a clear sense of purpose in life report lower levels of negative affect and less emotional reactivity to stressors in daily life (Hill *et al.,* 2018).

The evolutionary theory of loneliness posits that feeling lonely is an aversive biological signal that motivates the individual to repair or seek new social relationships, and leads to neural changes that impact attention and processing of social information (Cacioppo and Cacioppo, 2018). While our findings are in accordance with previous studies linking loneliness with altered RSFC in networks related to attention and executive control (Layden *et al.,* 2017), the results point to broader changes in brain connectivity across multiple networks. As with MIL, the most robust associations were observed for between network interactions, and specifically between the DN and FPN as well as networks implicated in more externally-directed cognition including attentional (e.g. VAN) or perceptual (e.g. SOM and visual networks) processing. While the breadth of these associations was not predicted, the VAN is associated with bottom-up or externally monitoring for behaviorally salient features of the environment (Corbetta and Shulman, 2002), presumably detected through connections with these perceptual systems. While we are unable to directly confirm this with the current data, this is consistent with behavioral accounts of hyper-vigilance for external social threat associated with loneliness. Further, the DN has been implicated in low mood and ruminative thoughts (DuPre and Spreng, 2018), which may be elevated by a sense of loneliness. However, several methodological considerations may account for differences between Layden *et al.* (2017) and the current findings. While a whole-brain analytic approach was used in both, we examined connectivity strength using individually-parcellated neurocognitive networks—thereby accounting for inter-subject functional connectivity variability—rather than focusing on standardized network parcellation schemes. Further, we used multivariate, data-driven analytical methods and a single model approach, including MIL whereas the earlier study focused on attention networks to test their hypotheses. Further, PLS methods allow for identification of both within and between network connectivity strengths in a single analytical model (McIntosh and Lobaugh, 2004; McIntosh and Mišić, 2013). Here, the between network associations were among the most robust, and most discriminating, patterns observed for loneliness and life meaning.

Our second hypothesis was based in part on recent findings that individual differences in both positive and negative behavioral traits have been associated with a unique configuration of intrinsic functional connectivity (Smith *et al.,* 2015). Specifically, increased connectivity within regions encompassing the DN was linked to positive behavioral traits such as life satisfaction, and inversely related to negative behavioral traits such as perceived stress (Smith *et al.,* 2015). Similarly, by including both loneliness and MIL in a single model, here we were able to identify a single pattern of functional connectivity implicating the DN that was associated with these positive and negative constructs. Connectivity within the DN, and its connections to the limbic network, were associated with a higher sense of life meaning and lower feelings of loneliness. In contrast, DN connectivity to externally-oriented attentional systems and cognitive control networks was associated with a higher sense of loneliness, and lower life meaning.

We further examined the features of whole-brain RSFC organization related to loneliness and MIL by interrogating the modular intrinsic network architecture. Increased modularity has been associated with more efficient processing operations and is generally considered to be a marker of brain health (Bullmore and Sporns, 2009; Wig, 2017). The intrinsic network organization of brain networks associated with loneliness was less modular as the DN and FPN were less differentiated from externally-directed attention and perceptual networks. As suggested above, this pattern of network dedifferentiation may reflect increased vigilance for social threat. Consistent with this idea, less modular brain network architecture has been associated with negative affect including depression, as well as normal and pathological aging (Andrews-Hanna *et al.,* 2014).

To our knowledge, this is the first study to investigate whole-brain patterns of RSFC associated with loneliness and MIL. Both MIL and loneliness are predictors of successful aging and an important future direction would be to examine how these patterns of intrinsic brain networks change in normal and pathological aging. Future examinations will also be necessary to explore how the connectivity patterns identified in the present study are dynamically shaped in response to task demands that require judgments of belonging and/or existential meaning. Further, MIL is distinct from meaning-seeking and meaning maintenance, and these differences will need to be explored with respect to loneliness and patterns of RSFC. This question is particularly relevant in light of past work demonstrating a distinction between the presence of meaning and the search for meaning (Heine *et al.,* 2006; Steger, 2012), and may have important implications for the interpretation of our results for loneliness given that the lack of belonging could both motivate or discourage an individual’s search for meaning.

By investigating associations between brain function, loneliness and MIL within a common analytical framework, we were able to identify a pattern of intrinsic functional connectivity that differentiated brain networks associated with higher MIL and lower loneliness from those associated with lower MIL and higher loneliness. Critically, between network interactions, particularly those involving the DN, were among the most robust and discriminating intrinsic network markers of loneliness and MIL. Behaviorally, these findings advance our understanding of these two constructs as distinct, yet interdependent, features of sociality (Stillman *et al.,* 2009; Lambert *et al.,* 2013; Stillman and Lambert, 2013). While speculative, the data also implicate the DN as a candidate network hub, suggesting that these brain regions may provide a neural conduit for shifting between feelings of isolation and purpose. If confirmed, these findings may inform future research to design behavioral and neural intervention strategies targeted at disrupting the reinforcing cycle of loneliness and life meaning.

## Conflict of interest:

The authors declare no competing financial interests.

## Supplementary Material

scan-18-359-File002_nsz021
