# Supplementary material for: Loneliness and meaning in life are reflected in the intrinsic network architecture of the brain
Source: Soc Cogn Affect Neurosci. 2019 Mar 29;14(4):423–33. doi: 10.1093/scan/nsz021 (PMC6523421; doi:10.1093/scan/nsz021)
Supplement: scan-18-359-File002_nsz021 [file scan-18-359-file002_nsz021.docx]

**Supplementary Material**

**Table 1**

*Means and SD of behavioral measures separated by gender*

|  | Female | Male |  |
| --- | --- | --- | --- |
|  | (Mean ± SD) | (Mean ± SD) | *t*-test (t, *p*) |
|  |  |  |  |
| Loneliness | 51.06 ± 8.08 | 50.87 ± 8.99 | (0.34, .732) |
| Meaning & Purpose | 52.62 ± 8.69 | 51.09 ± 8.71 | (2.70, .007) ** |
| Neuroticism | 17.15 ± 6.87 | 15.56 ± 7.75 | (3.33, .001) *** |
| Extroversion | 30.75 ± 5.93 | 30.71 ± 6.15 | (0.09, .925) |
| Agreeableness | 32.97 ± 4.64 | 31.13 ± 5.11 | (5.79, .001) *** |
| Conscientiousness | 35.17 ± 5.81 | 33.86 ± 5.94 | (3.43, .001) *** |
| Openness | 27.80 ± 6.04 | 28.94 ± 6.44 | (-2.81, .005) ** |
| Positive Affect | 50.44 ± 7.85 | 49.96 ± 7.81 | (0.94, .346) |
|  |  |  |  |

** *p*≤ 0.01; *** *p* ≤ 0.001, two-tailed


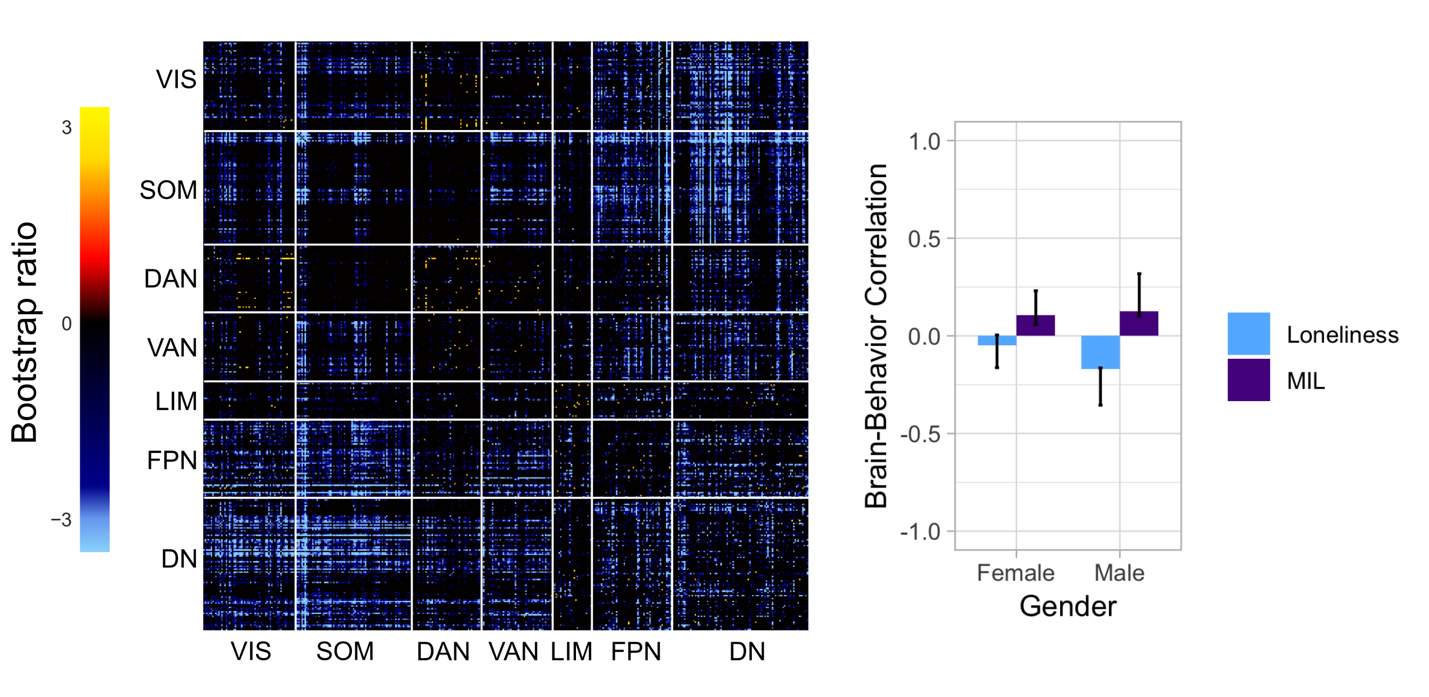


**Supplementary Figure 1.** Additional PLS analysis of loneliness and meaning in life (MIL) with gender. The figure displays the results for the first latent variable which identified a pattern of connectivity that reliably expressed the relationship between loneliness and MIL (permuted p = .006; 25% covariance explained) in both female (loneliness *r* = -0.05; MIL *r* = 0.10) and male (loneliness *r* = -0.17; MIL *r* = 0.13) participants. The error bars represent the 95% bootstrapped confidence interval. The matrix is thresholded to ±2 to 3.5 bootstrap ratio.
